# Supplementary material for: Relationships between maximum tongue pressure and second formant transition in speakers with different types of dysarthria
Source: PLoS One. 2022 Mar 8;17(3):e0264995. doi: 10.1371/journal.pone.0264995 (PMC8903301; doi:10.1371/journal.pone.0264995)
Supplement: Attachment — Submitted filename: Response_to_Reviewers_20211213.docx [file pone.0264995.s002.docx]

December 13, 2021,

Joerg Heber

Editor-in-Chief

Sara Finley, Ph.D.

Academic Editor

*PLOS ONE*

Dear Editor:

I wish to re-submit the manuscript, titled “Relationships between maximum tongue pressure and second formant transition in speakers with different types of dysarthria.” The manuscript ID is PONE-D-21-32058.

I thank you and the reviewers for your thoughtful suggestions and insights. The manuscript has significantly benefited from these insightful suggestions. I look forward to working with you and the reviewers to move this manuscript closer to publication in *PLOS ONE*.

The manuscript has been rechecked and the necessary changes have been made in accordance with the reviewers’ suggestions and marked in red. The responses to all the comments have been prepared and presented below.

Thank you for your consideration. I look forward to hearing from you.

Sincerely,

Toshiaki Tamura

Department of Speech, Language, and Hearing Sciences, Niigata University of Health and Welfare, Niigata city, Niigata 950-3198, Japan

Tel: +81 (25) 257-4507

Fax: +81 (25) 257-4507

Email: toshiaki-tamura@nuhw.ac.jp

**Point-by-point responses to comments from the Editor and Reviewers**

Thank you for reviewing our work. We appreciate all your comments and suggestions. We have revised the manuscript accordingly. Our point-by-point responses are presented below.

**Response to Journal Requirements**

**Comment 1:** *Please ensure that your manuscript meets PLOS ONE's style requirements, including those for file naming. The PLOS ONE style templates can be found at*

*https://journals.plos.org/plosone/s/file?id=wjVg/PLOSOne_formatting_sample_main_body.pdf and*

*https://journals.plos.org/plosone/s/file?id=ba62/PLOSOne_formatting_sample_title_authors_affiliations.pdf*

**Response 1: We are very grateful for your guidance. We have ensured that the manuscript meets the style requirements of PLOS ONE.**

**Comment 2:** *Thank you for stating the following financial disclosure:*

*“**This work was supported by the Japan Society for the Promotion of Science Grants-in-Aid for Scientific Research (KAKENHI) (https://www.jsps.go.jp/j-grantsinaid/) under Grant number JP20K19324.”*

*Please state what role the funders took in the study. If the funders had no role, please state: "**The funders had no role in study design, data collection and analysis, decision to publish, or preparation of the manuscript."*

*If this statement is not correct you must amend it as needed.*

*Please include this amended Role of Funder statement in your cover letter; we will change the online submission form on your behalf.*

**Response 2: Thank you for your valuable comment.** **Kindly revise the data availability statement accordingly for us. We have also added a statement to the cover letter.**

“This work was supported by the Japan Society for the Promotion of Science Grants-in-Aid for Scientific Research (KAKENHI) (https://www.jsps.go.jp/j-grantsinaid/) under Grant number JP20K19324. The funders had no role in the study design, data collection and analysis, decision to publish, and preparation of the manuscript.”

**Comment 3:** *We note that you have stated that you will provide repository information for your data at acceptance. Should your manuscript be accepted for publication, we will hold it until you provide the relevant accession numbers or DOIs necessary to access your data. If you wish to make changes to your Data Availability statement, please describe these changes in your cover letter and we will update your Data Availability statement to reflect the information you provide.*

**Response 3:** **The data required for the analysis of this paper are included in the supplementary data. Therefore, we did not provide data repository information. Please change the data availability statement accordingly for us. We apologize for the inconvenience. We have also added a request for change in the cover letter.**

“Furthermore, the data required for the analysis of this paper are included in the submitted supplementary data. Therefore, we did not provide the data repository information.”

**Comment 4:** *Please include captions for your Supporting Information files at the end of your manuscript, and update any in-text citations to match accordingly. Please see our Supporting Information guidelines for more information: http://journals.plos.org/plosone/s/supporting-information.*

**Response 4: We have added captions at the end of the manuscript according to the supporting guidelines. In addition, we have updated the citations in the text. New additions are shown in red.**

Page 37, Lines 59–596

Supporting information

S1 File. Dataset with all the participants. Re-measurement: result of a re-measurement by the same inspector > 6 months after the original measurement (30% of speakers with dysphoria).

Page 15, Lines 253–254

Details of all the participants, including healthy speakers, are shown in the S1 File.

**Response to the editor**

**Comment:** *I have received 2 reviewer reports for the paper, “Relationships between maximum tongue pressure and second formant transition in speakers with different types of dysarthria”. Both reviewers note that the research question is of potential interest to a wide range of scholars, and that the methods and interpretation of results are generally sound. However, both reviewers expressed some concerns related to the details of how the F2 slope was calculated. Given that this is the major contribution of this paper, I agree that more details need to be included in your revisions about how F2 slope was calculated, particularly the rationale for the method chosen. For example, Reviewer 2 asks questions about whether the F2 slope was calculated across the entire word, or within each vowel separately, so more details on how the vowels in the target words were analyzed distinctly from the consonants in the words is needed. An additional figure, as suggested by reviewer 1, would be welcome. Reviewer 1 also suggests several places where the writing could be improved for clarity, and I suggest following this advice, particularly where redundancies can be avoided (also noted by Reviewer 2), or jargon can be removed or explained (particularly in the abstract). Reviewer 2 also suggests that some analysis be conducted related to the type and severity level of dysarthria, which would be welcome, given the title of the paper.*

**Response: We appreciate the reviewer for these constructive comments. We agree with all your suggestions; we have tried to reduce redundancy, particularly in the Abstract section. The authors’ responses to the comments are as follows:**

**Reviewer #1:**

**General comments:**

*This paper has a new important issue on speech evaluation for dysarthria patients. But the outside reader of your manuscript it could be a little hard to understand correctly. Could you look at the points below:*

**Response: Thank you very much for your supportive comments and valuable suggestions for improving the quality of our manuscript.**

**Abstract**

**Comment 1: P3 L22-23**

*“The tongue pressure in speakers with dysarthria was significantly associated with the second formant transition slope.”*

*This sentence seems to have the same meaning as the one before this sentence. I recommend that you delete this sentence.*

**Response 1:** **Thank you for your helpful suggestion. We have omitted the duplication.**

**Comment 2: P3 L23-24**

*“This result suggests that the maximum isometric tongue strength is associated with* *articulation severity and tongue movement speed during articulation.”*

*The results showed that MTP was not correlated with speech intelligibility. The "the maximum isometric tongue strength is associated with articulation severity" in this sentence is inconsistent with the result.*

**Response 2: Our data did not show that the articulation severity was related to MTP. Therefore, we have omitted "articulation severity and" from the sentence as indicated.**

Page 3, Lines 37–38

This result suggests that the maximum isometric tongue strength is associated with tongue movement speed during articulation.

**Introduction**

**Comment 3: P5. L56-57**

*“these are qualitative assessments”*

*Does this* *“these” mean "Elevation strength of the anterior tongue"? If so, the elevation strength of the anterior tongue is quantitative assessment, not qualitative assessment.*

**Response 3: The word "these" meant "audibly articulatory precision and speech clarity." We have modified the sentence as follows:**

Page 4, Lines 67–68

However, since audibly articulatory precision and speech clarity are qualitative assessments, they affect the distribution of the data (ceiling or floor effect) [13].

**Comment 4: P3 L57**

*“they affect the distribution of data (ceiling or floor effect).”*

*Please add references.*

**Response 4: Thank you for your kind suggestion. We have cited the reference for this sentence as follows:**

Page 4, Lines 69

“they affect the distribution of data (ceiling or floor effect) [13].”

**Comment 5: P6 L67-69**

*“The hypothesis that … oral-DDK rates.”*

*Please add references.*

**Response 5: Thank you for your valuable suggestion. We have added the reference to this sentence:**

Page 5, Lines 79–81

The hypothesis that the tongue strength affects the speech speed does not appear to be supported by the weak correlation between the tongue strength and the articulation and oral-DDK rates [13].

**Material and Methods**

**Comment 6. P9 L121**

*“thickness, 0.5mm”*

*I think it would be more appropriate to write the diameter rather than the thickness of the bite block.*

**Response 1 : Thank you very much for your valuable suggestion. We have added the diameter of the bite block as follows:**

Page 8, Lines 131–133

The TPM-01 comprises a disposable probe, an injection tube as a connector, and a hard ring (bite block; length, 8.5 mm; thickness, 0.5 mm; diameter, 6.0 mm) device (Fig 1).

**Comment 2. P12 L177-179**

*I suggest you should make a figure for analysis methods about onset time, offset time, movement duration, and movement extent.*

**Response 2: Thank you very much for your valuable suggestion. We have created and included Figure 2 in our revised manuscript.**

Page 13, Lines 202–207

**Fig 2. A spectrogram of the word /taijo:/ produced by a man with stroke and mild unilateral upper motor neuron dysarthria.** This figure illustrates a major two vowel sequence and semivowel F2 movements in /ai/ and /jo/. The black lines running through the estimated centers of the first and second formants (F1 and F2) illustrate the formant tracing. The F2 slope was determined or the time interval, Duration, and the corresponding F2 frequency change, Extent.

**Comment 7. P12 L176-177**

*In the previous study, /aɪ/ was used to analyze the F2 movement. Why did you include /jo/ as well as /ai/ in the analysis in this study?*

**Response 7:** **Thank you very much for your pertinent comment. We apologize for not explaining this point accurately.**

**There is no consensus on the existence of double vowels in Japanese. In a previous study of a limited number of native Japanese speakers of dysarthria, vowel-to-vowel sequences were used (e.g. Tamura et al, 2021); however, there were concerns regarding the measurement errors. On the other hand, semivowels are also present in Japanese, and since we could obtain formant trajectories relatively close to the double vowels, we decided to add not only the /ai/ vowel sequence but also the semivowel /j/, which has characteristics similar to double vowels, to increase the data and reduce the measurement error. In addition, we included /jo/, which is different from /ai/, owing to the possibility that the opening and closing of the mandible could affect the formants (Yunusova et al, 2012). We have added the following explanations to our manuscript, which also address Comment 8.**

Page 11, Lines 190–192

F2 movement was measured based on a previous report measuring diphthong /aɪ/ [33] and semivowel /jæ/ [22].

Page 11–12, Lines 197–200

Based on a previous study of dysarthria [19], we averaged all the F2 slopes with different acoustic properties. This aimed to reduce the error from the actual syllable-specific tongue motion velocity, considering the possibility that mandibular opening and closing could be related to the context [22].

**Comment 8. P12 L184-185**

*The F2 slope values of /ai/ and /jo/ were calculated as one average value in this study. Why did you calculate the two types of two-vowels (/ai/ and /jo/) with different acoustic characteristics together as one average value?*

**Response 8: We wanted to minimize the error in the measurements, as shown in Response 7; therefore, we included /ai/ and /jo/, in our analysis, which have different acoustic properties, and both show a rapid rise and fall in F2. By mixing the measurement targets, we thought we could adjust the error of each measurement value. Kindly refer to our previous response.**

**Results**

**Comment 9. P14 L223-224**

*This cross-sectional study population included speakers with dysarthria who were admitted to acute and convalescent hospitals between September 2017 and June 2020. Please describe how many participants were entered, and how many participants did not match the inclusion criteria and were excluded, resulting in 65 participants matching the inclusion criteria. In addition, please describe the reasons for the exclusion.*

**Response 9: Thank you for your valuable suggestion. We have added the number of entries, the number of people excluded, and the reason for exclusion as follows:**

Page 14, Lines 249–250

There were 72 entries in this study. However, seven speakers with severe cognitive impairments were excluded.

**Comment 10. P14-15 L****225-227**

*“A significant difference in age was found between neurologically normal speakers and speakers with dysarthria (p < 0.001)”.*

*This sentence only showed the difference in age between neurologically normal speakers and speakers with dysarthria. Please describe the statistically analyzed differences in sex between neurologically normal speakers and speakers with dysarthria.*

**Response 10: Thank you for your helpful comment. The χ-square test showed a significant difference in the sex ratio between the groups. We have added the following sentence to the manuscript:**

Page 12–13, Lines 216–217

The χ-square test was used to evaluate the differences in the sex ratio.

Page 14–15, Lines 252–253

A significant difference in the age and sex was found between neurologically normal speakers and speakers with dysarthria (age: p < 0.001, sex: p < 0.031)

**Comment 11. P17 L244**

*“The average MTP of a speaker with dysarthria was 32.3 ± 9.9 kPa.”*

*The median of MTP was shown in table 2, so this statement did not need to show the mean of MTP.*

**Response 11: Thank you for pointing this out. We have omitted this sentence.**

**Discussion**

**Comment 12. P21 L303-304**

*“One of the factors behind these contradictory results is the inadequacy and imbalance of the subjects [10,13].”*

*Please add a specific explanation for “**inadequacy and imbalance of the subject”.*

**Response 12: Thank you for your helpful suggestion. It has long been pointed out that dysarthria occurs in a variety of disease backgrounds (i.e., Parkinson's disease, ALS, stroke) and that different diseases produce different speech symptoms (Darley et al., 1969; Duffy 2020). In addition, various organs are involved in speech production, and speech characteristics may also differ depending on the damaged site. In order to correctly interpret the results of this study, it is necessary to collect and analyze a large number of cases for each of these diseases and disorders. Speech is reportedly affected the most by the severe loss of tongue muscle strength (Neel et al, 2015, Solomon et al, 2017). We have added these statements to the manuscript:**

Page 23, Lines 358–362

Specifically, depending on the threshold of the tongue muscle weakness affecting speech, the correlation may not be clear when only a small number of patients has the most severe muscle weakness [11,13]. In addition, there may be a difference in the degree of contribution to the speech impairment between diseases in which muscle weakness is the main symptom [4,10] and other diseases [3,13].

**Comment 13. P21 L309-310**

*“Therefore, the results of this study support the weak correlation between the oral-DDK rate and tongue muscle strength.”*

*In this study, there was no significant correlation between the oral-DDK rate and tongue muscle strength. I consider that this statement is over-interpreted.*

**Response 13: Thank you for your suggestion. We agree with your opinion, and have modified the sentence as follows:**

Page 23, Lines 366–367

Therefore, the results of this study do not support a strong relationship between MTP and the oral-DDK rate.

**Comment 14. P21 L316-317**

*“This may have affected the results, as our study did not include participants with sustained combat injuries.”*

*Please describe how the non-inclusion of the participants with sustained combat injurie affected the results in this study.*

**Response 14: Thank you for your helpful suggestion. Solomon et al. (2017) included 16/55 people with combat injuries. A subgroup analysis with severely reduced tongue pressure also included 5/8 patients with brain injury as well as structural destruction of the orofacial region by blast explosion or gunshot wound and subnuclear paralysis. We believe that the orofacial disruption strengthens the relationship between the tongue muscle strength and speech function. Solomon et al. (2017) themselves mention a possible effect on the distribution in the combat injuries. Accordingly, we have added the following explanation:**

Page 24, Lines 374–376

Combat injuries with orofacial injuries may have a greater impact on the tongue function necessary for speech.

**Comment 15. P22 L321**

*were included →　were included* *in this study.*

**Response 15: Thank you for your valuable suggestion. The sentence has been revised as follows:**

Page 24, Line 377-379

Therefore, the difference in the correlation coefficient could not possibly detect a significant correlation because few participants with severe dysarthria were included in this study.

**Comment 16. P23 L354-355**

*“Therefore, the smaller effect size of the difference between the F2 slope and MTP suggests that speakers with mild dysarthria have lower discriminative function.”*

*This sentence is difficult to understand. Please describe why the smaller effect size of the difference between the F2 slope and MTP suggests that speakers with mild dysarthria have lower discriminative function.*

**Response 16: Thank you for your valuable suggestion. To clarify our intentions, we have revised the text as follows:**

Page 26–27, Lines 411–422

On the other hand, the effect size of the difference between the speakers with dysarthria and healthy participants on the MTP and F2 slope in this study was moderate. However, the effect of the difference in oral-DDK rate and speech intelligibility between healthy speakers and speakers with dysarthria was large. In a previous study, Japanese speakers with mild dysarthria (n = 16) showed no significant difference in the MTP compared with speakers without dysarthria (n = 29) [40]. In addition, the F2 slope was lower in speakers with moderate to severe dysarthria [21,41]. Thus, MTP and F2 slope are less capable in differentiating between healthy speakers and speakers with mild dysarthria than oral-DDK rate and speech intelligibility. Nevertheless, the findings of this study showed a moderate correlation between the F2 slope and MTP in speakers with dysarthria. The F2 slope is correlated with tongue muscle strength, which is stronger than the relationship between tongue muscle strength and other speech-related indicators.

**Comment 17. P24 L357**

*between the F2 slope and MTP. →　between the F2 slope and MTP* *in speaker with dysarthria.*

**Response 17: Thank you for your valuable suggestion. We have incorporated the following changes:**

Page 26, Lines 419－421

Nevertheless, the findings of this study showed a moderate correlation between the F2 slope and MTP in speakers with dysarthria.

**Comment 18.** *I consider that the oral-DDK rate and F2 slope are associated with the tongue movement speed during articulation. However, the MTP was significantly related to the F2 slope but not significantly related to the oral-DDK rate in this study. Please describe why the oral-DDK rate and F2 slope, which are associated with the tongue movement speed during articulation, had different results from each other.*

**Response 18: Thank you for your helpful suggestion. F2 slope reflects the rate of back-and-forth movement of the tongue during articulation. The DDK rate, on the other hand, is affected by the on/off of the voice and the opening/closing of the mandible. Moreover, the DDK rate does not take into account the distortion of the target articulation. Therefore, we believe that the F2 slope better reflects the motor function of the tongue during articulation.**

**We have added an explanation in the discussion section as follows:**

Page 27, Lines 423–434

In addition, the present study showed different results for the F2 slope and oral-DDK rate, which are related to tongue movement speed during articulation. This result is worthy of special mention. The DDK rate of /ta/ also depends on the speed of the voice on/off and mandibular opening/closing. In addition, the oral-DDK rate counts the number of syllables produced per second. Some articulation distortions (i.e., under shooting) are not reflected in the DDK rate measurements. Thus, both clear and unclear articulations are counted as one syllable (i.e., narrow or wide range of movement). The F2 slope, on the other hand, acoustically isolates the back-and-forth movement of the tongue during articulation and measures its speed. In a previous study, an oral-DDK task was not possible at maximum articulatory velocity [42]. Therefore, it is likely that the F2 slope better reflects the speed of the tongue movements than the oral-DDK rate, and this may have affected the results.

**Reviewer #2**

*This paper investigates the relationships between maximum tongue pressure and speech-related features, which include speech intelligibility, /ta/ DDK, and F2-slope. While speech intelligibility and /ta/ DDK were revealed to have no significant correlations with maximum tongue pressure, F2-slope was significantly correlated with the maximum tongue pressure (r=0.37, p<0.05). This paper proposes that the F2- slope may be useful at verifying the effect of tongue strength training.*

**Response: Thank you very much for your supportive comments and suggestions for the improvement of the quality of our manuscript.**

**Comment 1**: *Is the manuscript technically sound, and do the data support the conclusions?*

*- The idea of using F2-slope which can see both articulation accuracy and articulation rate, is convincing.*

*- F2-slope is usually used for analyzing diphthongs(one vowel), and sometimes vowel sequences. However, I am concerned about this experiment design, which analyze the F2-slope in a word level. Are the features extracted from the start of /a/ and end of /o/? If this is the case, the design should be revised in a major manner. Especially, for word /gaito/, the consonant is in between the two vowels, which must interrupt in extracting the F2-slope. Even for /taijo/, the difference of F2 between /a/ and /i/ are much larger compared to difference of F2 between /a/ and /o/. Analyzing the F2-slope for each vowel sequence/semi-vowel may be more persuasive.*

**Response 1: In this study, we did not analyze the whole word, but only the vowel-vowel transitions and semivowel (glide) portions. We analyzed three F2 slopes for each of these three locations: 1) the /ai/ transition in /taijo/, 2) the /jo/ glide in /taijo/, and 3) the /ai/ transition in /gaito:/. We have added the details of how we measured the F2 slope, as shown below, and created a measurement example in Figure 2.**

Page 11, Lines 188–190

Details of the measurement targets: 1) /taijo/'s /ai/ transition, 2) /taijo/'s /jo/ glide, and 3) /gaito:/'s /ai/ transition. Figure 2 shows an example of the /taijo:/ measurement.

Page 11, Lines 195–197

Therefore, the F2 slopes are expressed in absolute values (Hz/ms) throughout this manuscript to eliminate the positive/negative sign caused by the target-inherent F2-movement direction.

**Comment 2**: *Has the statistical analysis been performed appropriately and rigorously?*

*Mann-Whitney U test is applied to investigate the difference between healthy speakers and dysarthric speakers. Pearson Correlation is used to examine the relationships between MTP results and speech function features. Subgroup analysis by gender are appropriately performed.*

*Further subgroup analysis by dysarthric subtypes and severity levels should also be considered. In particular, the authors argue that the reason the study results do not agree with the previous results is because of the different distribution of speakers. Hence this analysis is necessary.*

**Response 2: Thank you for your helpful suggestion. We performed additional analyses grouped by dysarthria subtypes and by maximum tongue pressure. For the subtypes, there were significant correlations between F2 slope and MTP for Mixed and Flaccid, with Flaccid having the strongest correlations with oral-DDK rate and speech intelligibility compared with the other subtypes. In the group with the lower maximum tongue pressure, all the speech indices were significantly correlated with maximum tongue pressure.**

**We have added the following text to the Methods, Results, and Discussion section, respectively. Table 4 and Figure 4 have been added to the Results section.**

Methods

Page 13, Lines 224–225

In addition, a subgroup correlation analysis by the dysarthria subtype and MTP severity (divided into two groups by median) was performed.

Result

Page 20, Lines 311–318

　　　　　In the analysis by the dysarthria subtype, a significant correlation was observed between the MTP and F2 slope (Flaccid, rs = 0.786, p = 0.036; Mixed, rs = 0.640, p = 0.014; Table 4). Note that spastic (n = 3) and hyperkinetic (n = 1) types were excluded from the analysis due to their small sample size.

**Table 4. Spearman’s rank correlation coefficients (two sided) between the maximum tongue pressure and speech measures for each subtype of speakers with dysarthria (Total n = 59)**

Page 21–22, Lines 323–334

In the analysis by the groups categorized according to maximum tongue pressure, a significant correlation was observed between the MTP and all speech-related variables only in the lower MTP group (Speech intelligibility, rs = 0.397, p = 0.008; /ta/ DDK rate, rs = 0. 479, p = 0.038; F2 slope, rs = 0. 479, p = 0.038; Fig 4).

**Fig 4.** **Bivariate scatter plot and best-fit regression line of the maximum tongue pressure and speech-related variables in the two speakers with dysarthria groups divided according to the maximum tongue pressure by median**

Discussion

Page 25, Lines 388–390

　　　　　　The results of this study also showed that the correlation between the MTP and all the speech evaluations was stronger in the flaccid type, where muscle weakness was the main symptom, than in the other types.

**Comment 3:** *Have the authors made all data underlying the findings in their manuscript fully available?*

*- The datasheet used for the statistical analysis is uploaded. All features used in the analysis are included*

*- However, each notation must be explained of its meaning. For example, what does POST and typeNo imply?*

**Response 3: Thank you for your valuable suggestion. First, we changed "POST" to "****Re-measurement" in the supporting information file. We have also added an explanation for "** **Re-measurement." In addition, I deleted "typeNo" since it was not relevant information for the readers.**

Page 37, Lines 593–596

　　　　　S1 File. Dataset with all the participants. Re-measurement: Result of a re-measurement by the same inspector > 6 months after the original measurement (30% of speakers with dysarthria).

**Comment 4**: Further, each notations should be explained why each feature is included in the data. It is hard to understand why are height, weight, BMI, Albumin are included for the analysis.

**Response 4: Thank you for your valuable suggestion. The authors have reviewed the data and determined that the height, weight, BMI, and albumin do not need to be analyzed. Therefore, we have removed them from the data in the supporting information file.**

**Comment 5**: Brief information of dysarthric speakers should be stated in the manuscript. (subtypes)

**Response 5: Thank you for your valuable suggestions. We have made the following additions:**

Page 16, Lines 266–268

The breakdown of the dysarthria subtypes in the cases was as follows: spastic, 3; flaccid, 7; hypokinetic, 6; hyperkinetic, 1; ataxic, 7; unilateral upper motor neuron, 16; mixed, 14; and undetermined, 9.

**Comment 6:** 4**.** *Is the manuscript presented in an intelligible fashion and written in standard English?*

*- The manuscript is presented in an intelligible fashion and well-written in standard English.*

*- Though there are some redundant sentences that should be deleted for the final submissions.*

*Abstract is quite confusing, especially the last sentence : "However, based on the degree of these correlations, the hypothesis that the relationship between the maximum force of the tongue and speech function is weak is also strengthened." This sentence blurs the main idea of this paper, which suggests the significant correlation between maximum force of the tongue and F2-slope.*

**Response 6: Thank you for pointing this out. We have removed the aforementioned sentence and modified the entire abstract to correct the redundancy.**

**Comment 7: line 338 :** *F2-slope ->* *F2 (second formant)*

**Response 7: Thank you for your valuable suggestion. We have split the sentence into two and modified it as follows:**

Page 26, Lines 399–402

F2 roughly corresponds to the back-and-forth movement of the tongue [15]. The F2 slope, calculated from the movement duration and extent of the F2, is an acoustic index that correlates with the perceived accuracy of vowels [16].

**Comment 8: line 340 :** *It -> F2-Slope*

**Response 8: Thank you for your valuable suggestion. We have corrected it accordingly.**

Page 25–26, Lines 402–404

The F2 slope is speculated to reflect the speed of the tongue movement during articulation. In this study, the correlation between the F2 slope and tongue muscle strength was similar to all speakers with dysarthria in the sex-separated subgroups.
