# Supplementary material for: Relationships between maximum tongue pressure and second formant transition in speakers with different types of dysarthria
Source: PLoS One. 2022 Mar 8;17(3):e0264995. doi: 10.1371/journal.pone.0264995 (PMC8903301; doi:10.1371/journal.pone.0264995)
Supplement: Attachment — Submitted filename: Response_to_Reviewers_20220219.docx [file pone.0264995.s003.docx]

Manuscript number: PONE-D-21-32058

February 19, 2022,

Dr. Emily Chenette

Editor-in-Chief

Dr. Sara Finley

Academic Editor

*PLOS ONE*

Dear Editor:

Thank you for your e-mail regarding our manuscript titled “Relationships between maximum tongue pressure and second formant transition in speakers with different types of dysarthria.” The manuscript ID is PONE-D-21-32058R1.

We are grateful to you and the reviewers for your thoughtful suggestions and comments. The manuscript has significantly benefited from these insightful suggestions. I look forward to working with you and the reviewers to move this manuscript closer to publication in *PLOS ONE*.

The doi of references 19 and 41 in the reference list were incomplete and have been corrected. The retracted papers are not cited in this paper. Other minor corrections have also been made.

The manuscript has been revised and the necessary changes have been made in accordance with the reviewers’ suggestions; the revised sections are marked in red. We have also included point-by-point responses to the reviewer’s comments, which has been attached below.

We thank you again for the opportunity to revise our manuscript and hope this revised version will be suitable for publication in PLOS ONE. We look forward to hearing from you at your earliest convenience.

Sincerely,

Toshiaki Tamura

Department of Speech, Language, and Hearing Sciences, Niigata University of Health and Welfare, Niigata city, Niigata 950-3198, Japan

Tel: +81 (25) 257-4507

Fax: +81 (25) 257-4507

Email: toshiaki-tamura@nuhw.ac.jp

**Point-by-point responses to comments from the Editor and Reviewers**

Thank you for reviewing our work. We appreciate all your comments and suggestions. We have revised the manuscript accordingly. Our point-by-point responses are presented below.

**Response to Journal Requirements**

**Comment 1:** *Please review your reference list to ensure that it is complete and correct. If you have cited papers that have been retracted, please include the rationale for doing so in the manuscript text, or remove these references and replace them with relevant current references. Any changes to the reference list should be mentioned in the rebuttal letter that accompanies your revised manuscript. If you need to cite a retracted article, indicate the article’s retracted status in the References list and also include a citation and full reference for the retraction notice.*

**Response 1: Thank you for your suggestion.** **The doi of references 19 and 41 in the reference list were incomplete and have been corrected. The retracted papers are not cited in this paper. Other minor corrections have also been made.**

**Response to the editor**

**General comments:**

*Both original reviewers have read the revised manuscript and believe that their concerns have been met in the revision. However, both reviewers have minor suggestions for clarification and wording. These are important in order to be sufficiently clear for a broad audience. I also have some minor comments related to clarity and wording:.*

**Response: We appreciate the reviewer for their constructive comments.** **The authors’ responses to the comments are as follows:**

**Comment 1: Line 129**: *"Comprised 20 participants" should be 'comprised of 20 participants"*

**Response 1:** **Thank you for the detailed confirmation. Our manuscript was submitted for professional English editing and proofreading, and we were informed that “comprised of” is a grammatically incorrect phrase. We hope that retaining the original phrase here will not be an issue..**

Taken from the Merriam-Webster website:

“Although comprised of is an established standard for "being composed or constituted of," it is often liable to criticism and scrutiny. The correct version put forward by grammar guides is to use "composed of" or "comprises" such as "the cake is composed of flour and eggs" or "comprises flour and eggs."

**Comment 2: Line 139:** *"highly guaranteed" seems somewhat superlative. I would suggest toning this down, or noting that the measurement is believed to be reproducible and reliable*

**Response 2:** **Thank you for your helpful suggestion. Accordingly, we have made the following revision in the sentence:**

Page 8, Line 139

The reproducibility and reliability of this device has been validated in a previous study [26].

**Comment 3: Lines 225-226**: *"The F2 was determined or the..." This sentence is somewhat confusing to read. Please make sure that capitalization is consistent.*

**Response 3: Thank you for your helpful suggestion. We have made the following revision in the sentence:**

Page 13, Lines 225-226

Duration is the time interval of the F2 movement. Extent is the range of frequency change in the F2 movement.

**Comment 4: Line 344:** *"Bold value indicates and" This sentence is confusing. It seems that a word is missing here. Value should be 'values'?*

**Response 4:** **Thank you for your helpful suggestion.** **We have made the following revisions in the text:**

Page 22, Line 344: Values in bold and with asterisks indicate that rs is significant (p < 0.05) (two-tailed).

**Comment 5: Line 357:** *Make sure to have consistent capitalization for 'Lower'.*

**Response 5: Thank you for your helpful suggestion. Accordingly, we have removed the capitalization from the sentence:**

Page 23, Lines 357-359

　　　　Speakers with lower maximum tongue pressure and dysarthria (lower group) tended to have lower speech-related variables, such as lower maximum tongue pressure.

**Reviewer #1:**

**General comments:**

*This paper has been properly revised from the first manuscript. But a part of your manuscript remain a little hard to understand correctly. Could you look at the point below:*

**Response: Thank you for your supportive comments and valuable suggestions for improving the quality of our manuscript. The authors’ responses to the comments are as follows:**

**Material and Methods**

**Comment 1: P13 L221-225**

*“Fig 2. A spectrogram of the word…. corresponding F2 frequency change, Extent.”*

*In Figure 2, the analysis methods of F2 onset and F2 offset for /ai/ and /jo/ were unclear. Please explain the detailed definition of F2 onset and F2 offset for /ai/ and /jo/.*

**Response 1:** **Thank you for comment. We have adjusted the sound spectrogram in Fig. 2 to improve clarity regarding the whole picture. We have also made the following revisions in the text:**

Page 12, Lines 205–212

The F2 linear predictive coding tracks on a wide-band spectrogram (analysis bandwidth, 300 Hz) were manually edited and identified. The 20/20 rule (specifying a frequency change of ≥20 Hz during 20 ms in the transition onset and offset) was applied [41]. If the F2 track was unclear due to hoarseness or other noises, we identified it by referring to the changes in F1 (opening and closing of the mandible) that were almost synchronized with F2 in the /ai/ and /jo/ sequences. Thus, relatively stationary portions of the vowel before or after the target transition were not included in the analysis.

Page 13, Lines 226–228

The F2 movement for each syllable is determined by a change in frequency of at least 20 Hz during a 20 ms period, not including the relatively stationary portions before and after the syllable.

**Comment 2: P12 L225-226**

*The F2 slope was determined or the time interval, Duration, and the corresponding F2 frequency change, Extent.*

*→ The F2 slope was determined or the time interval, duration, and the corresponding F2 frequency change, extent.*

**Response 2: Thank you for your helpful suggestion.** **We have revised this for more clarity.**

Page 13, Lines 225-226

Duration is the time interval of the F2 movement. Extent is the range of frequency change in the F2 movement.

**Reviewer #2**

**General comments:**

*The authors have adequately addressed the given comments raised in a previous round of review. I feel confident that the manuscript has become much clearer. The manuscript is technically sound, and the data support the conclusions. The statistical analysis has been performed appropriately and rigorously (Mann-Whitney U test - assessment of the difference between dysarthric speakers vs healthy speakers; Pearson correlation - correlations between MTP and speech-related features (speech intelligibility, /ta/ DDK, F2-slope) within speakers with dysarthria. However, there are some minor comments for the improvements of clarity.*

**Response: Thank you for your supportive comments and suggestions for the improvement of the quality of our manuscript.** **The authors’ responses to the comments are as follows:**

**# Abstract**

**Comment 1: line 31**: *Some speakers with dysarthria -> Speakers with dysarthria*

*- The paper reports the significant difference between dysarthric speakers and healthy speakers. To state as 'some speakers' seems unnecessary.*

**Response 1: Thank you for your helpful suggestion.** **We have incorporated your suggestion in our paper.**

**Comment 2**: **line 39**: *This result suggests that the maximum isometric tongue strength is associated with tongue movement speed during articulation.*

*- oral DDK is also known to represent the tongue movement speed during articulation. Elaborating on the difference between oral DDK and F2-slope may help the readers better understand the paper's main contribution.*

**Response 2: Thank you for your helpful suggestion.** **We have made the following revision in the sentence:**

Page 3, Lines 37–39

The oral diadochokinesis rate, which is related to the speed of articulation, is affected by voice on/off, mandibular opening/closing, and range of motion. In contrast, the second formant slope was less affected by these factors.

**# Introduction**

**Comment 3:** **line 51-53**: *does speech clarity align with speech intelligibility? Please elaborate on this terminology. It is difficult to relate 'severity' with speech-related indicators (compared to articulation rate, oral-DDK rate). Please elaborate.*

**Response 3:** **Thank you for your question.**

**-Yes, speech clarity is the same as speech intelligibility. For more clarity, the term “speech intelligibility” has been used consistently in the paper. In addition, “severity” has been deleted because it includes impressions other than speech.** **We have made the following revision in the sentence:**

Page 3, Lines 51–53

However, current reviews have reported no significant relationship between tongue strength and speech-related indicators, such as speech intelligibility, articulation rate, and oral-DDK rate [6–8].

**Comment 4: line 63**: *overall severity (of dysarthria? muscle strength?)*

**Response 4: Thank you for your comment. Overall severity is an auditory measure of dysarthria, and corresponds to a comprehensive assessment of speech intelligibility and naturalness. We have added the following to the sentence to make it clearer:**

Page 4, Lines 61–64

Additionally, speakers with dysarthria in whom tongue muscle strength is lower than the lower limit of normal speakers have moderate-to-severely reduced articulatory precision and overall severity (including speech intelligibility and naturalness) [13].

**Comment 5**: **line 65:** *what does /ta/<5.8 syllables/s infer? Stating the oral-DDK rate for healthy speakers or mild dysarthria is necessary.*

**Response 5: Thank you for your valuable suggestion. We have made the following additions:**

Page 4, Lines 66–67

In a previous cross-sectional study [13], dysarthria speakers (n = 8) with severe anterior tongue elevation muscle strength had an oral-DDK rate of <5.8 syllable/s for the syllable /tʌ/. In contrast, 44.6% of the remaining speakers with dysarthria had an oral-DDK rate of >5.8 syllables/s.

**Comment 6: line 68, line 72:** *please explain what speech audibility is. The authors are using 3 terminologies in the manuscript - speech* *intelligibility,* *speech clarity, speech audibility.*

**Response 6:** **Thank you for pointing this out. We were using these terms in the same context. Therefore, we have unified all three terms into “speech intelligibility.”**

Page 3, Lines 53; Page 4, Lines 68, 72; Page 5, Lines 73

**Comment 7:** **line 76 :** *F2 slope -> F2* *(second formant)*

**Response 7: Thank you for the detailed confirmation. We have defined the second formant as F2 in the sentence preceding this one(line 74). We hope that retaining the original phrase here will not be an issue..**

**Comment 8: line 82-83:** *relatively slow changes in tongue shape. Please elaborate. How is tongue shape related to the decrease in the F2-slope? In addition, tongue shape seems not to be related to movement speed, which is the essence of this paper.*

**Response 8: Thank you for your comment. The tongue is a muscle without any joints. Therefore, “relatively slow changes in tongue shape” has the same meaning as “slow movement of the tongue.”** **In other words, the back-and-forth motion of the tongue during articulation is slower, resulting in a smaller F2 slope.** **To make it clearer, we have added the following sentence.**

Page 5, Lines 81–85

The clear explanation for the decrease in the F2 slope in speakers with dysarthria is the relatively slow changes in tongue shape [22]. Specifically, the back-and-forth motion of the tongue during articulation is slower and/or the range of movement is narrower, resulting in a longer and thinner change in the F2 movement.

**# Materials and Methods - Participants**

**Comment 9:** Information of dysarthric speakers (how many speakers, Gender, Age, etc) is omitted.

**line 120:** please account for the reason why height, weight, albumin, BMI are collected for this study.

**Response 9: Thank you for your valuable suggestion.** **These factors were considered to account for the possible effects on tongue muscle strength and speech caused by factors other than the primary disease causing dysarthria.** **The following sentences have been added to the manuscript.**

Page 7, Lines 122–124

　　　　These factors were considered to account for the possible effects on tongue muscle strength and speech caused by factors other than the primary disease causing dysarthria.

**# Materials and Methods - Oral-DDK rate**

**Comment 10: line 183:** *3s of the central parts: Please explain why the authors decided to use only the central parts of the audio, rather than the full audio.*

**Response 10: Thank you for your valuable suggestion.** **We used a 3-second duration of the middle part of the audio to reduce the effects of speech irregularities during speech onset and respiratory dysfunction during the second half of the task. In the syllable repetition task, the first syllable is often uttered longer than the following syllable in normal subjects (Ackerman et al, 1995). In speakers with dysarthria, it is possible that the initial effect is due to a freezing at movement onset (e.g., hypokinetic type). In addition, speakers with dysarthria are more likely to be affected by breath-holding in the second half of oral-DDK due to reduced respiratory function. The following sentences have been added to the manuscript.**

Page 11, Lines 183–186

　　　　To reduce the effects of speech irregularities such as freezing, slurring, or syllable prolongation during speech onset, or respiratory dysfunction during the second half of the task, we extracted ~3 s of recorded data from the middle parts of the audio for the analysis.

**Comment 11: line 204:** *Specific explanation of how the authors determined the onset and offset of F2 is needed for replication.*

**Response 11: Thank you for your comment.** **Based on your suggestion, I added the method of determining the onset and offset.**

Page 12, Lines 205–212

The F2 linear predictive coding tracks on a wide-band spectrogram (analysis bandwidth, 300 Hz) were manually edited and identified. The 20/20 rule (specifying a frequency change of ≥20 Hz during 20 ms in the transition onset and offset) was applied [34]. If the F2 track was unclear due to hoarseness or other noises, we identified it by referring to the changes in F1 (opening and closing of the mandible) that were almost synchronized with F2 in the /ai/ and /jo/ sequences. Thus, relatively stationary portions of the vowel before or after the target transition were not included in the analysis.

**# Materials and Methods - Second formant transition**

**Comment 12:** *fig 2 - fast formant > first formant*

**Response 12:** **Thank you for pointing this out. We have corrected this.**

**# Results**

**Comment 13: line 322-323 :** *Correlation between MTP & speech intelligibility, MTP & /ta/ DDK by sex groups should also be presented by gender.*

**Response 13:** **Thank you for your valuable suggestion.** **We have added the following sentence:**

Page 20, Lines 324-326

The correlation between MTP and speech intelligibility was significant only in males (rs = -0. 328, p = 0.030). There was no significant correlation between MTP and the /ta/ DDK rate in either sex.

**Comment 14: line 335- 337:** *Descriptions for the analysis of other subtypes are missing. (hypokinetic, ataxic, etc.)*

**Response 14: Thank you for pointing this out.** **We have added the following sentence:**

Page 21, Lines 337–338

There was no significant correlation between any of the combinations in the other subtypes.

**# Discussion**

**Comment 15: line 362-373:** *The fact that the authors analyzed the correlations between MTP and speech-related indicators within dysarthric speakers does not stand out in this paragraph.*

**Response 15: We have added the following sentence:**

Page 23, Lines 363–364

In this study, we measured the tongue pressure in speakers with various types of dysarthria, following which, we conducted a correlation analysis between MTP and speech-related indicators..

**Comment 16: line 414–417:** *According to the results, speech intelligibility and oral-DDK measurements did not show significant correlations with MTP.*

**Response 16: Thank you for pointing this out. We have made the following revision:**

Page 26, Lines 414–417

　　　　In our results, speech intelligibility and oral-DDK rate were not significantly correlated with MTP in all subtypes. However, the flaccid type (n = 7) had the highest correlation coefficient among all types (speech intelligibility, rs = -0.436; /ta/ DDK rate, rs = 0.517). Additional subtype-specific studies are warranted.

**Comment 17: line 432:** *some speakers with dysarthria - please concretely describe the corresponding speakers.*

**Response 17: Thank you for your valuable suggestion.** **We have reconsidered the results of this study and believe that it is not possible to determine the specific characteristics of the speakers. Therefore, we have deleted “some” and modified the sentence as follows:**

Page 27, Lines 432–435

In summary, in speakers with dysarthria, tongue strength may be associated with tongue movement velocity during articulation. Our findings partially support the hypothesis that “muscle weakness is associated with slow speech” [2].

**# Conclusion**

**Comment 18: line 483:** *the appropriateness of articulation and speed*

**Response 18: Thank you for your suggestion. We have added this to the text.**
